# Development of a method for isolating brain capillaries from a single neonatal mouse brain and comparison of proteomic profiles between neonatal and adult brain capillaries

**DOI:** 10.1186/s12987-023-00449-w

**Published:** 2023-06-23

**Authors:** Yudai Hamada, Seiryo Ogata, Takeshi Masuda, Shingo Ito, Sumio Ohtsuki

**Affiliations:** 1 Department of Pharmaceutical Microbiology, School of Pharmacy, Kumamoto University, 5-1 Oe-Honmachi, Chuo-Ku, Kumamoto, 862-0973 Japan; 2 Department of Environmental Medicine and Molecular Toxicology, Tohoku University Graduate School of Medicine, 2-1 Seiryo-Machi, Aoba-Ku, Sendai, 980-8575 Japan; 3 Department of Pharmaceutical Microbiology, Graduate School of Pharmaceutical Sciences, Kumamoto University, 5-1 Oe-Honmachi, Chuo-Ku, Kumamoto, 862-0973 Japan; 4 Department of Pharmaceutical Microbiology, Faculty of Life Sciences, Kumamoto University, 5-1 Oe-Honmachi, Chuo-Ku, Kumamoto, 862-0973 Japan

**Keywords:** Brain capillaries, Isolation, Neonate, Proteomics, Single brain, Age, Transporter, Extracellular matrix

## Abstract

**Background:**

The functions and protein expressions of the blood–brain barrier are changed throughout brain development following birth. This study aimed to develop a method to isolate brain capillaries from a single frozen neonatal mouse brain and elucidate the enrichment of brain capillaries by quantitative proteomic analysis. We further compared the expression profile of proteins between neonatal and adult brain capillary fractions.

**Methods:**

The brain capillary fraction was prepared by the optimized method from a single frozen mouse neonatal brain on postnatal day 7. The brain capillary fractions and brain lysates were digested by trypsin and analyzed by liquid chromatography-mass spectrometry for quantitative proteomics.

**Results:**

By optimizing the isolation method, we observed brain capillaries in the fraction prepared from a single neonatal mouse brain (nBC fraction). A protein amount of 31.5 μg, which is enough for proteomic analysis, was recovered from the nBC fraction. By proteomics analysis, the brain capillary selective proteins, including Abcb1a/Mdr1, Slc2a1/Glut1, Claudin-5, and Pecam-1, were found to be concentrated > 13.4-fold more in nBC fractions than in whole brain lysates. The marker proteins for neurons and astrocytes were not concentrated in nBC fractions, while those of pericytes and microglia were concentrated. Compared to adult mouse brain capillary fractions (aBC fractions), the expressions of Abcb1a/Mdr1a, Abcc4/Mrp4, and Slc2a1/Glut1 were significantly lower in nBC fractions than in aBC fractions, whereas those of Slc1a4/Asct1, Slc1a5/Asct2, Slc7a1/Cat1, and Slc16a1/Mct1 were significantly higher. Amino acid transporters, Slc38a5/Snat5, showed the greatest nBC-to-aBC ratio among transporters (9.83-fold). Network analysis of proteins expressed differentially between nBC and aBC fractions revealed that the proteins with terms related to the extracellular matrix were enriched.

**Conclusions:**

We succeeded in isolating brain capillaries from a single frozen brain of a neonatal mouse at postnatal day 7. Proteomic analysis revealed the differential expression in brain capillaries between neonatal and adult mice. Specifically, amino acid transporters, including Slc1a5/Asct2 and Slc38a5/Snat5, were found to be induced in neonatal brain capillaries. The present isolation method will promote the study of the function and expression of the neonatal blood–brain barrier.

**Supplementary Information:**

The online version contains supplementary material available at 10.1186/s12987-023-00449-w.

## Background

Brain capillary endothelial cells (BCECs), together with pericytes, astrocytes, neurons, and extracellular matrix as a neurovascular unit, form the blood–brain barrier (BBB) [1]. BCECs express tight junction proteins to form tight junctions, restricting paracellular diffusions between the blood and brain [2]. BCECs also express various transporter proteins supplying nutrients from the blood to the brain and limiting drug distribution to the brain [3]. Receptor proteins expressed in the BCECs regulate the functions of the BBB and mediate the transport of ligand molecules across it [4].

The functions and protein expressions of the BBB are changed during brain development after birth. P-glycoprotein is an efflux transporter selectively expressed in BCECs and limits drug entry into the brain [3]. The expression of P-glycoprotein in rat brains increases at postnatal week 8 compared to that at week 2, and the brain distribution of oseltamivir, a P-glycoprotein substrate, simultaneously decreases [5]. An age-dependent increase in P-glycoprotein staining intensity has been reported in the human cortex [6]. The expression of the glucose transporter GLUT1 in brain capillaries has also been reported to increase from neonates to adults in mice and rats [7, 8]. The influx rate of glucose into the brain of 15–18-day-old rats was half that of adults aged 9–12 weeks [9]. In contrast, the expression of the transporter for lactate and ketone bodies, MCT1, is higher in the brain capillaries of neonates than in those of adults [7, 8]. The influx rates of lactate and pyruvate into the brain were also greater in neonatal rats than in adult rats [9], indicating that the developmental changes in the BBB are important for understanding the regulation of homeostasis during brain development and the distribution of central nervous system-acting drugs in the brain during childhood.

Isolated brain capillaries are the samples essential for elucidating BBB transport mechanisms during development. Single-cell analysis has recently been used to analyze gene expression in well-characterized brain cells, including BCECs [10, 11]. However, single-cell analysis is limited to gene expression, and isolated brain capillaries are necessary for functional and protein expression analyses. The preparation of brain capillary fractions is difficult for neonatal brains than for adult brains because of the former’s smaller volume and increased fragility [12]. In a previous report, brain capillaries were isolated from the brains of 12–15 newborn mice [13]. Another study isolated the brain capillaries of 50 mice at postnatal day 5 and conducted proteomic and transcriptomic analyses [14]. Brain capillaries were isolated from the brains of 4–15 neonatal rats and 2 adult rats [8]. Recently, we developed a method to efficiently isolate brain capillaries from the frozen brain of an adult mouse using a bead homogenizer, cell strainer, and glass beads [15]. Through this method, we prepared brain capillary fractions with higher enrichment and recovery than that of the standard isolation method, suggesting that brain capillaries can be isolated from the brain of a neonatal mouse using our method.

Therefore, this study aimed to develop a method to isolate brain capillaries from a single frozen neonatal mouse brain and elucidate the enrichment of brain capillaries by quantitative proteomic analysis. Brain capillaries were isolated from the frozen brain of a neonatal mouse on postnatal day 7, and brain capillary enrichment in the isolated fraction was evaluated using proteomics. We further compared the expression profile of the proteins in the neonatal and adult brain capillary fractions. The changes in protein expression between neonatal and adult brain capillaries were comparable to those reported in previous studies, and amino acid transporters, including Slc38a5/Snat5 and Slc1a5/Asct2, were upregulated in neonatal brain capillaries compared to those in adult brain capillaries. We also observed changes in the expression of proteins related to the extracellular matrix, such as laminins, collagens, and integrins, in neonatal brain capillaries.

## Methods

### Animals

C57BL/6 J pregnant mice were purchased from Japan Clea (Tokyo, Japan). All animals were bred in the Kumamoto University Faculty of Pharmaceutical Sciences Animal House and were housed in 12-h light/dark environment. All animal experiments were approved by the Institutional Animal Care and Use Committee at Kumamoto University and followed the Fundamental Guidelines for Proper Conduct of Animal Experiments and Related Activities in Academic Research Institutions under the jurisdiction of the Ministry of Education, Culture, Sports, Science, and Technology as well as the Animal Research: Reporting in Vivo Experiments guidelines.

### Brain capillary isolation

Newborn mice were dissected at 7 days of age. A single neonatal mouse brain was frozen in liquid nitrogen in a 1.5-mL tube and stored at − 80 °C. For preparation, a single mouse brain was transferred to a 2-mL screw-cap tube (Watson, Tokyo, Japan) after thawing, and 1 mL of a homogenizing buffer (101 mM NaCl, 4.6 mM KCl, 2.5 mM CaCl_2_, 1.2 mM KH_2_PO_4_, 1.2 mM MgSO_4_, 15 mM HEPES, pH 7.4) was added. The brain was subsequently homogenized by a bead homogenizer (Bead Mill 4, Thermo Fisher Scientific, Waltham, MA, USA) for 30 s at 1 m/s without beads to reduce homogenizing power. Thereafter, the homogenate was uniformly homogenized by pipetting and transferred to a new 2-mL tube. Moreover, 50 μL of the brain homogenate was dispensed in another tube as whole brain lysate. The homogenate was centrifuged (1000 × *g*, 10 min, 4 °C), and the supernatant was carefully removed. Up to 1 mL of the homogenizing buffer was added to the pellet and suspended by pipetting gently. After suspension, an equal volume of 32 w/v% dextran/homogenize buffer was added into the tube and mixed by inverting. The samples were immediately centrifuged (4500 × *g*, 15 min, 4℃), and the supernatant was collected into a new 2-mL tube. The pellets were kept on ice. The supernatant was centrifuged (4500 × *g*, 15 min, 4℃) again, and the supernatant was discarded. After removing the fat content adhered at the wall of the tube using Kimwipe (Nippon Paper Crecia, Tokyo Japan), pellets were suspended in the suspension buffer (homogenized in buffer containing 25 mM of NaHCO_3_, 10 mM of glucose, 1.2 mM of pyruvate, and 5 g/L of bovine serum albumin, 200 μL × 2), and the samples of the two tubes were combined into one tube. A cell strainer (pluriStrainer-Mini, 70 μm mesh, pluriSelect, Leipzig, Germany) was filled with 800 mg of glass beads (0.35–0.5 mm, AS ONE, Osaka, Japan) and washed 4 times with 500 µL of suspension buffer. Then, the combined suspension sample was added to a cell strainer with glass beads and washed 10 times with 500 µL of suspension buffer. Glass beads were transferred to a new tube using a spatula. Thereafter, 1 mL of suspension buffer was added to glass beads and mixed by inverting. The supernatant was then quickly transferred into a new tube. The suspension buffer of 500 µL was re-added on glass beads and mixed by inverting; the supernatant was quickly transferred into the previous tube. The tube was centrifuged (3300 × *g*, 5 min, 4℃), and the supernatant was removed. The pellet was suspended using 100 µL of a homogenization buffer. Part of the isolated brain capillary fraction was used for microscopy. Isolated brain capillary fractions were lysed in a hypotonic buffer (10 mM NaCl, 1.5 mM MgCl_2_, 10 mM Tris–HCl, pH 7.4) by sonication, and protein levels were measured using a Pierce bicinchoninic acid (BCA) protein assay kit (Thermo Fisher Scientific).

### Western blot analysis

A western blot analysis of the isolated brain capillary fraction was performed as previously described [16]. Samples were separated on a sodium dodecylsulfate (SDS) polyacrylamide gel and blotted onto a polyvinylidene fluoride (PVDF) membrane. The following primary antibodies were used: Claudin-5, 1/4000 dilution (35–2500; Thermo Fisher Scientific); β-actin, 1/10000 (8H10D10; Cell Signaling Technology, Danvers, MA, USA); and horseradish peroxidase (HRP) conjugated secondary antibodies, 1/10000 dilution (Goat anti mouse IgG HRP, 7076S, Cell Signaling Technology). Band intensity was quantified using ImageJ software (National Institutes of Health, Bethesda, MD, USA).

### Quantitative proteomic analysis

The peptide samples of brain capillary fractions and whole brain lysates for quantitative proteomics were prepared by trypsin digestion using phase transfer surfactant. Proteomic analysis was conducted as described previously [17, 18]. Briefly, each sample was analyzed by data independent acquisition (DIA/SWATH) on the TripleTOF 6600 (SCIEX, Framingham, MA, USA) interfaced with the Eksigent nanoLC 400 (SCIEX). Mass calibration and checking of intensities, retention times, and peaks were done every 5–6 samples by trypsin-digested β-galactosidase with the auto-calibration function in Analyst TF 1.7.1 (SCIEX). Protein identification and quantification were conducted using the library-free search function in DIA-NN 1.8 with the UniProt mouse reference proteome allowing one miss-cleavage [19]. Default settings were used for amino acid modifications and the properties of the precursors and fragments. The intensities of the precursors were normalized using retention time-dependent cross-run normalization, and the concentration of each protein was calculated from specific peptides using the MaxLFQ algorithm [20], which was integrated into the DIA-NN. Peptides and proteins were filtered at a false discovery rate of less than 1% for identification and quantification. To validate the quality of the present proteomic data, the distribution of intensities and coefficients of variance (CV) of the identified proteins are shown in Additional file 2: Fig. S1. The score plot of principal component analysis is shown in Additional file 2: Fig. S2. The intensity distributions were not significantly different among the samples according to a one-way ANOVA (Additional file 2: Fig. S1A and B). The %CV histograms overlapped between the two or four groups, and the averages and medians were less than 21.0% and 17.7%, respectively (Additional file 2: Fig. S1C and D). The replicate samples from each group formed clusters in the score plots (Additional file 2: Fig. S2). The protein concentrations of all quantified proteins are listed in Additional file 1: Tables S1 and S2. Raw data files of the liquid chromatography with tandem mass spectrometry analysis have been deposited in jPOST (jPOST ID: JPST001978/PXD039296 for neonatal data and JPST002139/PXD041770 for adult data).

### Statistical analysis

Numerical data are expressed as mean ± standard deviation. Between-two group comparisons were performed using the paired-samples t-test or Welch t-test in Microsoft Excel (Microsoft Corp.; Redmond, WA, USA). Network analysis of the differentially expressed proteins was conducted using STRING 11.5 (https://string-db.org/) [21]. The UniProt accession number list of differentially expressed proteins was set to search for multiple proteins, and the organism was set as *Mus musculus*. Clustering was performed using MCL with an inflation parameter of 3 (Fig. 5A and Additional file 2: Fig. S3). Cluster 1, which contained 60 proteins, was further analyzed using functional enrichment analysis (Fig. 5B and Additional file 2: Fig. S4, Additional file 1: Table S4). Principal component analysis was performed using SIMCA 14 (Sartorius, Gottingen, Germany) with centering and scaling variables set to Pareto Variance. Graphs and heat maps were created using GraphPad PRISM7 (GraphPad, Boston, MA, USA).

## Results

### Isolation of brain capillaries from a single neonatal mouse brain

**Fig. 1:**
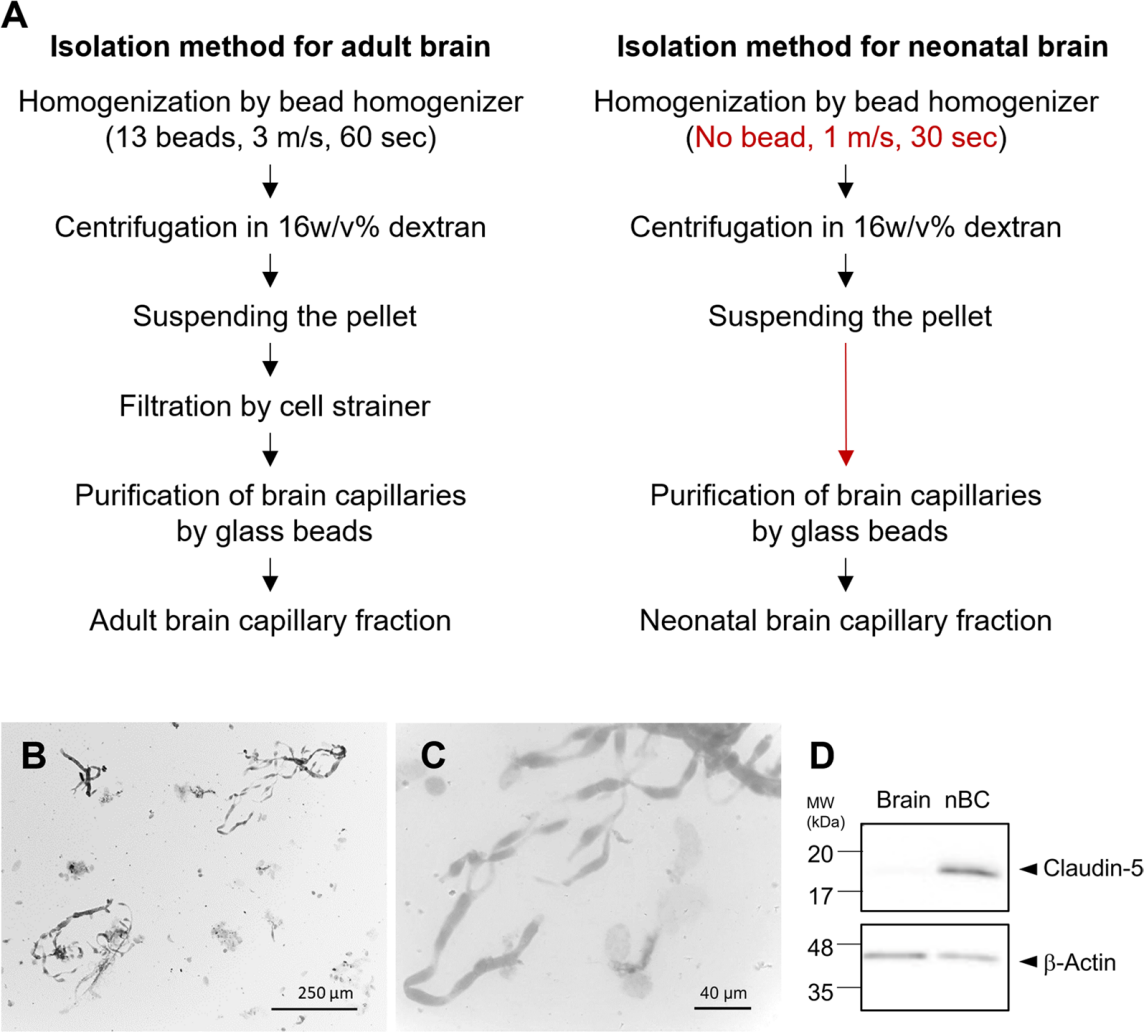
Isolation procedure and enrichment of brain capillaries from a single neonatal mouse brain. **A** Isolation procedures of brain capillaries from the single frozen brain of adult and neonatal mice. The modifications in neonates are indicated in red. **B** and **C** Images of the isolated brain capillary fractions from a single neonatal mouse brain with different magnifications. Scale bar, 250 µm in **B** and 40 μm in **C**. **D** Western blot analysis of Claudin-5 in whole brain lysate and neonatal brain capillary (nBC) fraction

First, we prepared brain capillary fractions from neonatal mice brains using the previously reported isolation method from the single brain of an adult mouse [15]. However, no brain capillary was observed in the recovered fraction. To adapt to the fragility of the neonatal brain, the isolation method was modified by reducing homogenizing power, and filtration was omitted to improve recovery (Fig. 1A). Consequently, the fraction enriching neonatal brain capillaries (nBC fraction) was obtained from a single neonatal brain (postnatal day 7), as shown in Fig. 1B and C. The diameter of the isolated capillaries was 5–10 μm as previously reported [22]. The enrichment of brain capillaries was examined by Claudin-5, which is specifically expressed in BCECs (Fig. 1D). Claudin-5 was detected in the nBC fraction, but not in the whole brain lysate, suggesting that brain capillaries were concentrated in the nBC fraction.

### Comprehensive analysis of isolated neonatal brain capillary fractions by quantitative proteomics

**Fig. 2:**
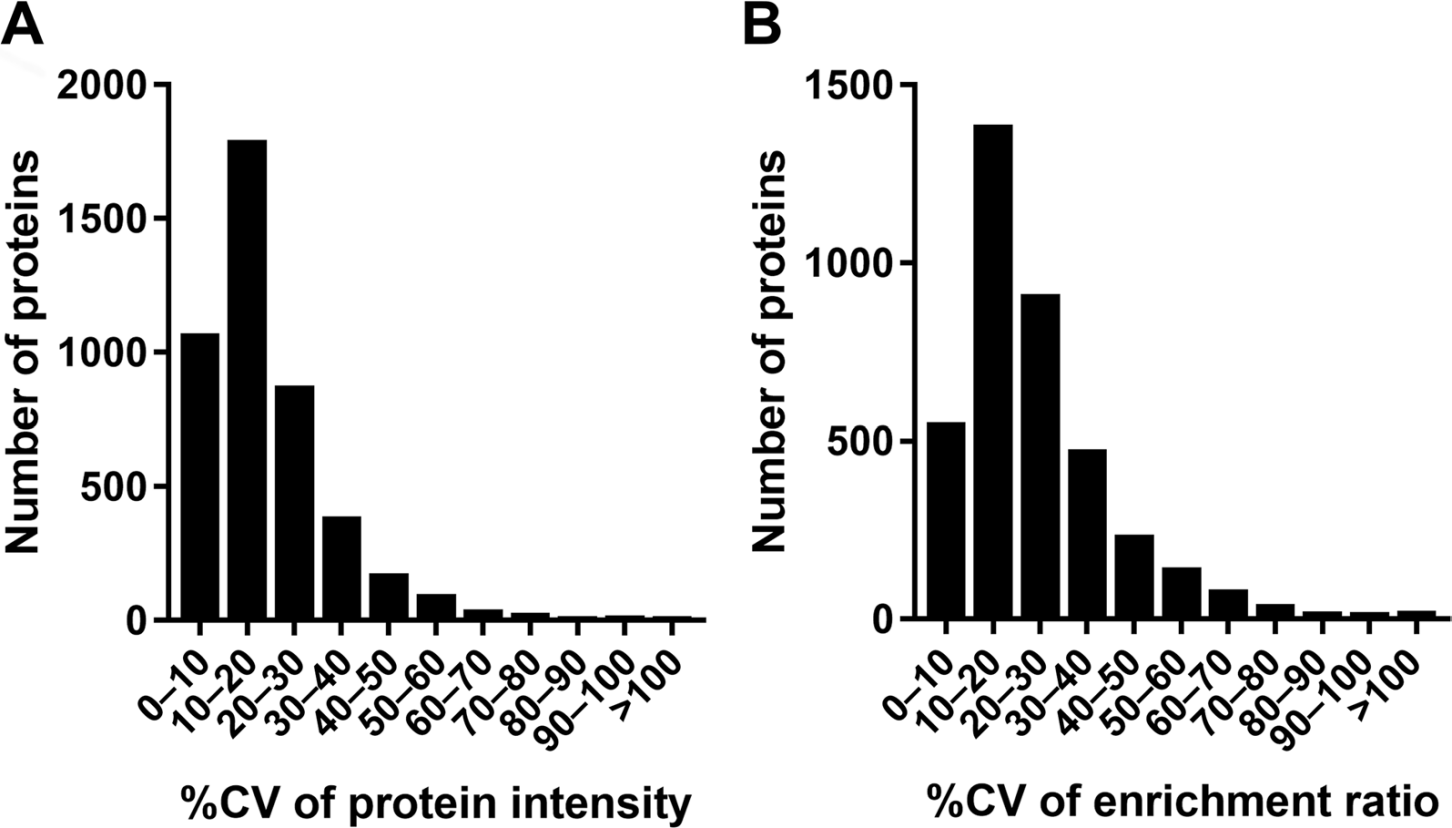
Reproducibility of brain capillary isolation from a single neonatal mouse brain. **A** Histogram of the coefficient of variance (%CV) of protein intensities of 4,362 proteins detected in the nBC fractions (n = 4). **B** Histogram of the %CV of the nBC-to-brain enrichment ratios of 3,782 proteins detected in both nBC fractions and whole brain lysates (n = 4)

The enrichment of brain capillaries in the nBC fraction was further evaluated by proteomics. The nBC fraction was prepared from a single neonatal brain by four biological replicates. The total protein amount of the fraction was 31.5 ± 9.7 μg (n = 4), which is less than half of the fraction from a single adult mouse brain (73.5 μg) [15] but sufficient for proteomic analysis. The analysis identified 4,362 proteins in the nBC fraction and 5,090 proteins in whole brain lysate; 3,782 proteins were identified in both fractions, and 2,739 proteins demonstrated significant differences (*p* < 0.05, Additional file 1: Table S1). The reproducibility among replicates was evaluated by the coefficient of variance (%CV) of the quantified values of the 4,362 proteins identified in the nBC fraction. The average %CV was 18.9%, and the 75^th^ percentile was 23.8% (Fig. 2A). The reproducibility of the enrichment process was assessed by the %CV of nBC-to-brain enrichment ratio (Fig. 2B). The average %CV of the enrichment ratio was 23.8%, and the 75^th^ percentile was 30.1%, suggesting sufficient reproducibility in the preparation of the nBC fraction for analyzing the protein expression profile.

The enrichment of proteins selectively expressed in brain capillaries was investigated to assess the enrichment of brain capillaries in the nBC fraction (Table 1). The nBC-to-brain enrichment ratios of the selective proteins in the brain capillary were greater than 5.23, with a maximum value of 20.8. The enrichment ratio of Claudin-5 was 17.5, and its concentration in the nBC fraction is consistent with the result of the western blot shown in Fig. 1D. Enrichment ratios were also calculated for the marker proteins of neurons, astrocytes, pericytes, oligodendrocytes, and microglia (Table 1). The enrichment ratios of markers for neurons, astrocytes, and oligodendrocytes were less than 1, suggesting the exclusion of those cells during the capillary isolation from the brain. In contrast, the enrichment ratios of the markers for pericytes and microglia were significantly greater than 1, indicating the inability to remove pericytes and microglia from the nBC fraction. The range of the ratio of pericyte markers was similar to that of brain capillary markers (5.51–16.2 vs. 5.93–20.8), while that of the microglia maker was lower (about 2). Therefore, pericytes were enriched in the fraction in a manner similar to that in brain capillaries, and the enrichment of microglia was lower than that of pericytes.

**Table 1:** Enrichment ratios of proteins in the nBC fraction to the whole brain lysate

Protein	nBC-to-brain enrichment ratio		
	Mean	SD	Significance
Brain capillary endothelial cells			
﻿Abcb1a/Mdr1a	17.6	5.5	***
Abcc4/Mrp4	8.85	13.63	***
Abcg2/Bcrp	6.75	1.77	***
Slc2a1/Glut1	20.1	4.5	***
Slc7a5/Lat1	5.23	0.79	***
Slc22a8/Oat3	6.63^a^		
Slco1c1/Oatp1c1	11.5	3.6	***
Tie2/Tek	nBC only		
Tfr1/Tfrc	18.0	4.4	***
Claudin-5	17.5	7.6	***
Occludin	nBC only		
JAM1	5.93	1.61	***
Esam	15.3	7.7	***
Pecam1	13.4	8.7	***
vWF	20.8	17.9	***
Neurons			
Synaptophysin	0.0861	0.0131	***
Map2	0.141	0.023	***
Astrocytes			
Gfap	0.885	0.235	
Slc1a2/Eaat2	0.192	0.021	***
Slc1a3/Eaat1	0.510	0.0495	***
Pericytes			
Pdgfrb	16.2	9.0	***
Muc18/Mcam	5.51	0.51	***
Oligodendrocytes			
Cnp	0.275	0.021	***
Microglia			
Progranulin	2.05	0.23	***
Cdk11b	2.03	1.32	***
Vascular smooth muscle cells			
Acta2	3.05	1.04	*
Taglin	1.02^a^		
Myh11	6.80	2.56	***

Each nBC-to-brain enrichment ratio represents the mean and standard deviation (SD) (n = 4), *p < 0.05 and ***p < 0.001 represent significant difference between the neonatal brain capillary (nBC) fraction and whole brain lysate assessed by the paired-samples t-test. nBC only, protein expression was detected only in the nBC fraction. ^a^Detected in all four nBC fractions, but only in two of four whole brain samples

Contamination of the cerebral artery and venous vessels was assessed using proteins selectively expressing vascular smooth muscle cells that adhere to large cerebral blood vessels [23]. The enrichment ratios of aortic smooth muscle actin (Acta2), transgelin (Tagln), and myosin-11 (Myh11) ranged from 1.02 to 6.80 (Table 1), which were lower than those of the marker proteins for BCECs or pericytes. Therefore, the arteries and venous vessels were retained in the nBC fraction but were not as concentrated as the brain capillaries and pericytes.

### Proteomic comparison of brain capillary isolation between neonatal and adult brain capillary fractions

**Fig. 3:**
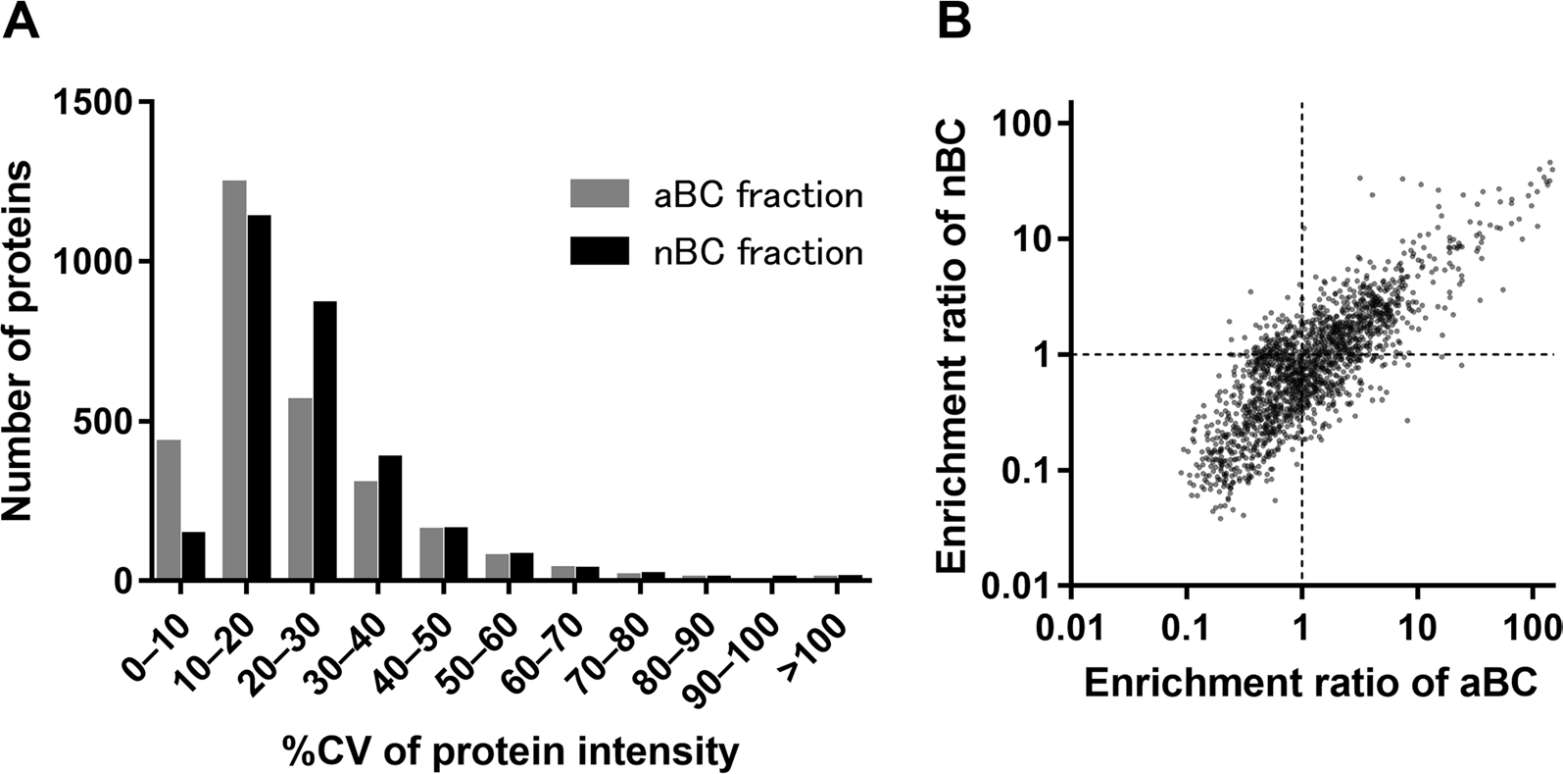
Comparison of the reproducibility and enrichment between neonatal and adult brain capillary fractions. **A** Histogram of the coefficient of variance (%CV) of the protein intensities of 2,816 proteins, which were detected in both the neonatal brain capillary (nBC) and adult brain capillary (aBC) fractions (n = 4). **B** Correlation of the BC-to-brain enrichment ratios between the nBC and aBC fractions. The correlation was compared for 2,155 proteins, which were identified in all brain capillary (BC) fractions and whole brain lysates (n = 4)

To evaluate changes in protein expression in mouse brain capillaries between neonates and adults, we compared the proteomic data of brain capillary fractions isolated from the single brain of a neonatal mouse and those of an adult mouse (nBC and aBC fraction, respectively). The proteomic data of the aBC fraction reported in our previous study was re-analyzed in concomitance with the present nBC fraction data [15]. Consequently, 3,673 and 3,680 proteins were identified in the nBC and aBC fractions, respectively, and 2,816 proteins were identified in both fractions (Additional file 1: Table S2). The number of identified proteins decreased compared to that of the analysis in the last section as the mass spectrometry data obtained from different measurements were compared. To assess the reproducibility of preparation between the nBC and aBC fractions, the %CV values of the intensity of the common 2,816 proteins were compared. The average %CV value of the nBC fraction was significantly greater than that of the aBC fraction (19.3% vs 16.5%, *p* < 0.001, Fig. 3A). However, the distribution of %CV values were similar between both fractions, suggesting that the reproducibility of preparation was slightly lower in the nBC fraction than in the aBC fraction, but the variation was small for comparison of protein expression. Moreover, the brain capillary (BC)-to-brain enrichment ratios of 2,155 proteins, which were identified in all brain capillary fractions and all brain lysate samples, were compared. As shown in Fig. 3B, the enrichment ratios were significantly correlated between neonatal and adult brains (r = 0.832, *p* < 0.0001), suggesting that the enrichment manners are similar in the fractions prepared from neonatal and adult brains.

### Comparison of protein expression between neonatal and adult brain capillary fractions

We compared the expression levels of proteins involved in the transport and cell junctions at the BBB between the nBC and aBC fractions (Table 2). Transporters, Abcb1a/Mdr1a, Abcc4/Mrp4, Abcg2/Bcrp, Slc2a1/Glut1, Slc3a2/4F2hc and Slco1a4/Oatp1a4 were significantly less expressed, while Slc1a4/Asct1, Slc7a1/Cat1, Slc16a1/Mct1 and Slco1c1/Oatp1c1 were significantly more expressed in the nBC fraction than in the aBC fraction. Among receptors, transferrin receptor were 4.11-fold more expressed in the nBC fraction than in the aBC fraction, while FcRn in the nBC fraction were expressed at 48.4% of that in the aBC fraction. As tight and adherens junction proteins, the expression of Claudin-5 was 1.49 higher in the nBC fraction than in the aBC fraction, whereas occludin, JAM-1 and Cadherin-5/VE-cadherin expression in the nBC fraction significantly decreased to 25.8%, 46.1%, and 35.1%, respectively, of that in the aBC fraction.

**Table 2:** Ratio of protein expression in nBC fraction to aBC fraction

Protein	nBC-to-aBC ratio		
	Fold	SD	Significance
ABC transporters			
Abcb1a/Mdr1a	0.189	0.060	***
Abcc4/Mrp4	0.247	0.065	***
Abcg2/Bcrp	0.471	0.072	***
SLC transporters			
Slc1a4/Asct1	3.94	0.32	***
Slc2a1/Glut1	0.128	0.149	***
Slc3a2/4F2hc	0.763	0.106	**
Slc7a1/Cat1	1.80	0.11	***
Slc7a5/Lat1	1.09	0.15	
Slc16a1/Mct1	2.48	0.14	***
Slc22a8/Oat3	1.13	0.23	
Slc27a1/Fatp1	0.910	0.261	
Slco1a4/Oatp1a4	0.159	0.066	***
Slco1c1/Oatp1c1	1.54	0.20	**
Receptors			
Transferrin receptor	4.11	0.30	***
Insulin receptor	0.804	0.228	
Lrp1	1.01	0.07	
FcRn large subunit	0.484	0.064	***
Tight junction and adherens junction proteins			
Claudin-5	1.49	0.27	*
Occludin	0.258	0.111	***
Jam1	0.461	0.079	***
Cadherin-5/VE-cadherin	0.351	0.089	***

Each nBC-to-aBC enrichment ratio represents the mean and standard deviation (SD) (n = 4). *p < 0.05, **p < 0.01, and ***p < 0.001 represent significant differences between the neonatal brain capillary (nBC) and adult brain capillary (aBC) fractions assessed by the Welch t-test

**Fig. 4:**
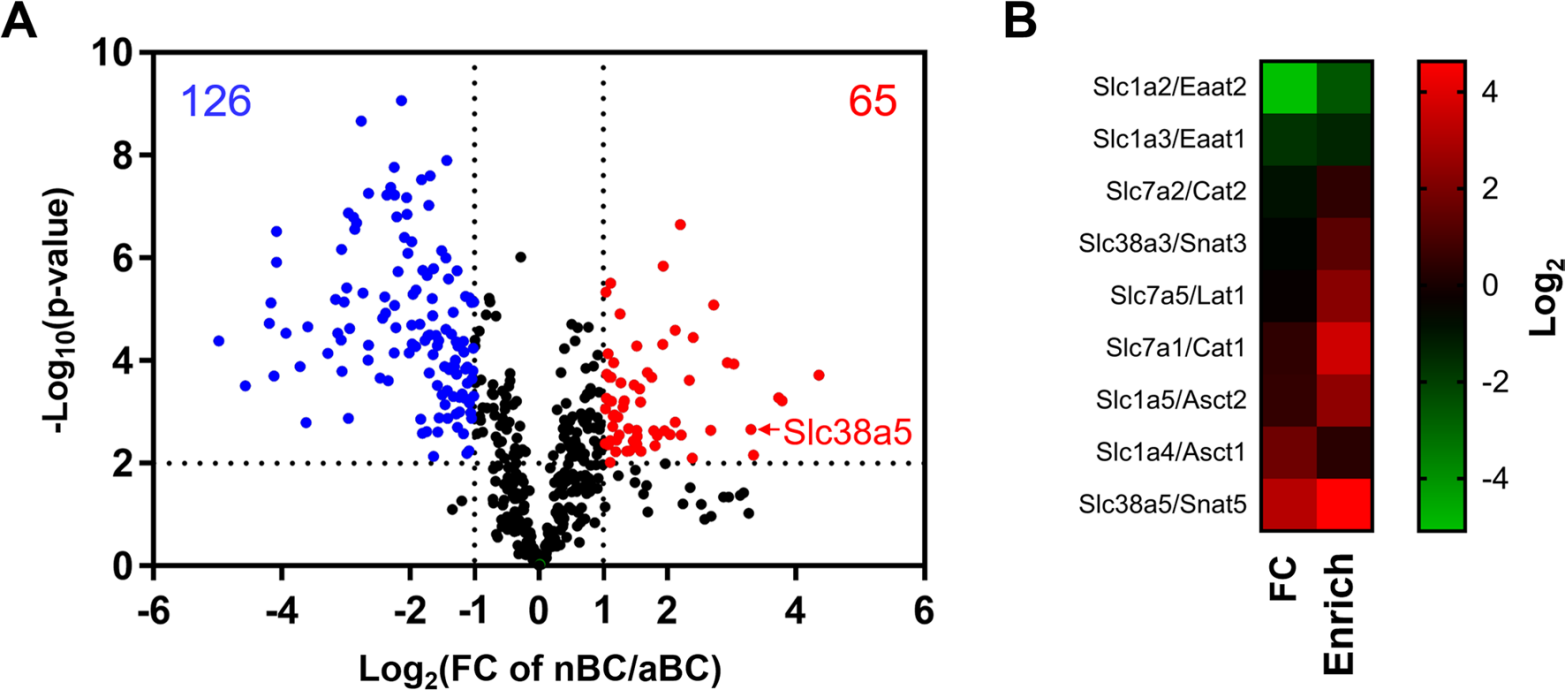
Differential expression of proteome and amino acid transporters between neonatal and adult brain capillary fractions. **A** Volcano plot of the average expressions of 504 brain capillary (BC)-enriched proteins compared between the neonatal brain capillary (nBC) and adult brain capillary (aBC) fractions (n = 4). BC-enriched proteins were extracted by the BC-to-brain enrichment ratio greater than 5 in either the nBC or aBC fraction. **B** Heatmaps of fold changes and nBC-to-brain enrichment ratios of the amino acid transporters detected in the nBC and aBC fractions. Heatmap on the left indicates fold changes in the nBC-to-aBC fraction protein expression ratios (FC). Heatmap on the right indicates the nBC-to-brain enrichment ratios (Enrich). The fold change and ratio are indicated by log﻿_2_.

To perform a comprehensive comparison, we extracted the proteins with a BC-to-brain enrichment ratio greater than 5 in either the nBC or aBC fractions as the BC-enriched proteins, because the proteins selectively expressed in brain capillaries had a BC-to-brain enrichment ratio greater than 5.23 (Table 1). Consequently, 504 proteins were extracted, and 191 proteins showed a significantly different expression between fractions, with an nBC-to-aBC ratio > 2 or < 0.5, and a *p*-value < 0.01 (Fig. 4A, Additional file 1: Table S3). Among the proteins significantly more expressed in the nBC fraction than in the aBC fraction, Slc38a5/Snat5, a sodium-coupled neutral amino acid transporter 5, showed the greatest nBC-to-aBC ratio among transporters (9.83-fold, Fig. 4A). Therefore, by focusing on amino acid transporters, 9 proteins were detected in the nBC and aBC fractions, including non-BC-enriched proteins (Fig. 4B). Slc7a1/Cat1, Slc1a5/Asct2, Slc1a4/Asct1, and Slc38a5/Snat5 were > 1.8-fold more expressed in the nBC fraction than in the aBC fraction. The nBC-to-brain enrichment ratios of these transporters were all > 5.51, except for that of Slc1a4/Asct1. The expressions of Slc7a2/Cat2, Slc38a2/Snat3 and Slc7a5/Lat1 were not significantly different between the fractions. The expressions of Slc1a2/Eaat2 and Slc1a3/Eaat1 were significantly < 0.386-fold lower in the nBC fraction than in the aBC fraction, with nBC-to-brain enrichment ratios of < 0.506.

**Fig. 5:**
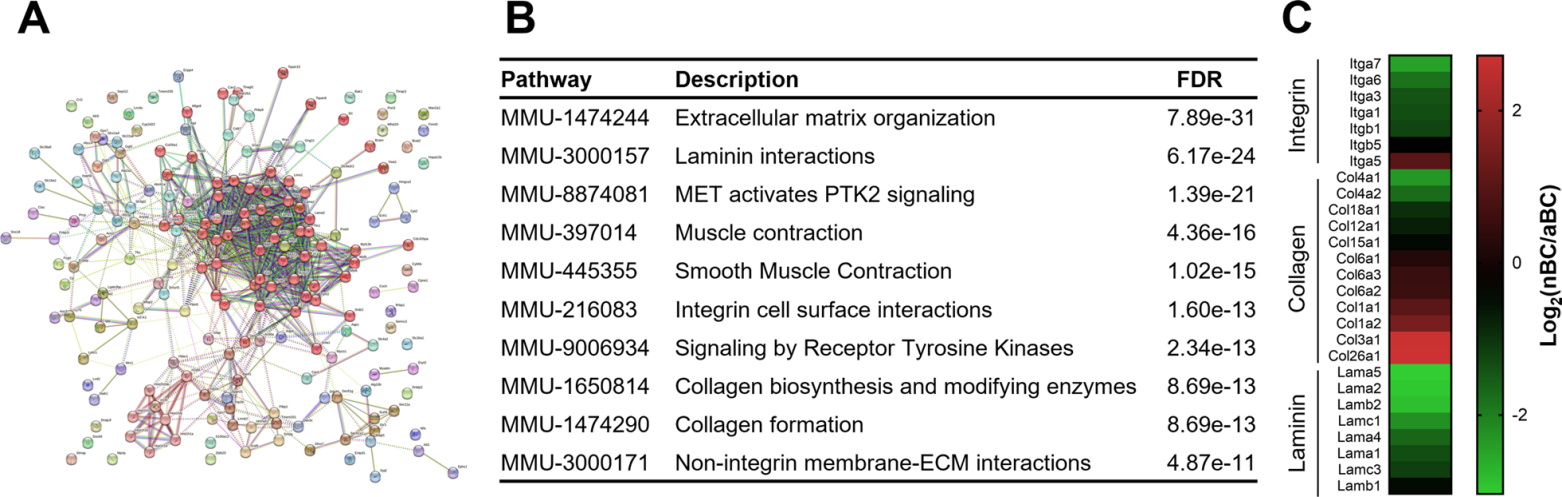
Network analysis of differentially expressed proteins and differential expression of extracellular matrix proteins between fractions. **A** Network map of 191 differentially expressed brain capillary (BC)-enriched proteins by STRING. The proteins indicated by the red circle constitute the largest network group by clustering analysis. The enlarged maps were indicated in Additional file 2: Figs. S3 and S4. **B** Pathways enriched in the largest network cluster containing 60 proteins indicated by the red circle in Fig. 5A. Pathways from the reactome are shown. Pathways from gene ontology and KEGG are shown in Additional file 1: Table S4. **C** Heatmaps of fold changes in BC-enriched extracellular matrix proteins compared between the neonatal brain capillary (nBC) and adult brain capillary (aBC) fractions. The fold is indicated by log_2_. FDR, false discovery rate

Network analysis was conducted with 191 proteins showing significantly different expression between the nBC and aBC fractions in Fig. 4A. Consequently, 60 proteins were classified into one large cluster (red symbols in Fig. 5A; details are shown in Additional file 2: Figs. S3 and S4). In this cluster, terms related to the extracellular matrix, laminin, integrin, and collagen were enriched (Fig. 5B). The protein expression levels of integrins and laminins were lower in the nBC fraction than in the aBC fraction (Fig. 5C). In contrast, the expressional changes were different among collagen subtypes: Col26a1 and Col3a1 were > 6.37-fold more expressed in the nBC fraction than in the aBC fraction, while Col4a1 and Col4a2 were < 0.330-fold less expressed in the nBC fraction than in the aBC fraction. These results suggest that extracellular components around brain capillaries are different between neonates and adults.

## Discussion

We developed an isolation method for brain capillaries from a single frozen neonatal brain. Due to the fragility of the neonatal brain, brain capillaries cannot be recovered through the standard homogenization performed for adult brains. The present modifications are critical for the recovery of capillaries from the neonatal brain (Fig. 1). Quantitative proteomics validated the enrichment of brain capillaries in the isolated fraction, as well as the sufficient reproducibility to perform proteome comparisons.

Brain cell contamination is unavoidable for brain capillary isolation. The nBC fraction contained enriched pericytes, and neurons were efficiently removed (Table 1). This was similar to the aBC fraction [15]. Enrichment of the identified proteins was significantly correlated between the nBC and aBC fractions (Fig. 3B). This suggests that the overall enrichment and exclusion of brain cells in the nBC fraction were similar to those in the aBC fraction. However, the contaminations of astrocytes were different between the nBC and aBC fractions. The astrocyte marker Gfap was 4.10-fold more enriched in the aBC fraction than in whole brain lysate [15], while it was not significantly concentrated in the nBC fraction (0.89-fold, Table 1). It has been reported that astrocytes are not as densely projecting as that at adult age compared to that at 2 weeks of age, and that clear astrocyte boundaries have not been established [24]. Thus, the adhesion of astrocytes to brain capillaries may be immature, suggesting that they were not enriched in association with neonatal brain capillaries.

Contamination should be considered when interpreting protein expression in the brain capillary fraction. For example, the expression of Na^+^/K^+^ATPase β1 and α3 (Atp1b1 and Atp1a3, respectively) in the nBC fraction was 12.4% and 12.8%, respectively, of that in the aBC fraction, indicating a lower expression of these proteins in nBC (Additional file 1: Table S2). However, the nBC-to-brain enrichment ratios were 0.0781 and 0.0562, and the aBC-to-brain ratios were 0.253 and 0.237 for Atp1b1 and Atp1a3, respectively. This suggests that Atp1b1 and Atp1a3 are not dominantly expressed in either nBC or aBC, and their expression in the brain capillary fraction contains residual expression in neurons and/or microglia. A previous immunohistochemical study reported that Atp1b1 was expressed in neurons and glial cells, and Atp1a3 was expressed in neurons of postnatal day 19 mouse brains [25]. This report also demonstrated a lower expression of both proteins in neonatal mouse brains than in adult brains. Since the expression of proteins that were not enriched in the brain capillary fraction was largely influenced by their expression in non-brain capillary cells, the present comprehensive comparison of protein expression in the brain capillaries between neonates and adults was performed using BC-enriched proteins (Figs. 4 and 5).

The previous report has compared protein expressions in isolated rat brain capillary fractions at 14 and 56 days of age by targeted proteomics [8]. In that report, brain capillaries were isolated from 4–15 neonatal rats and 2 adult rats. It is worth comparing the protein expression changes between our present study and those in a previous report to validate the reproducibility of brain capillary isolation in neonatal and adult mouse brains. In both studies, Abcb1a/Mdr1a, Abcc4/Mrp4, and Abcg2/Bcrp were 0.2–0.4-fold less expressed in neonatal brains than in adult brains. Furthermore, 18 proteins were quantified in both studies, and the nBC-to-aBC ratio was significantly correlated (r = 0.882, p < 0.0001; Additional file 2: Fig. S5). Therefore, the current isolation method can be applied to compare the expression and function of brain capillaries between neonate and adult mice, and the proteomic data in the present study is helpful for the screening of the expressional changes of proteins at the BBB between neonates and adults.

The present proteome comparison between the nBC and aBC fractions suggests that altered expression of amino acid transporters occurred in neonatal brain capillaries (Fig. 4B). Slc1a4/Asct1 and Slc7a1/Cat1 were significantly more expressed in the nBC fraction than in the aBC fraction. The previous immunohistochemical analysis demonstrated a higher expression of these amino acid transporters in neonatal brain capillaries in mice and rats [26, 27]. To our knowledge, this proteomic analysis is the first to suggest that the expressions of the amino acid transporters, Slc38a5/Snat5 and Slc1a5/Asct2, are induced in neonatal brain capillaries (Fig. 4B). The functional role of Slc38a5/Snat5 at the BBB has not been fully elucidated. However, in the brain RNA-seq database, its mRNA was selectively expressed in endothelial cells in both mouse and human brains [28, 29]. Slc1a5/Asct2 has been reported to mediate the L-isomer selective efflux transport of aspartate in adult rat brains [30]. Slc38a5/Snat5 and Slc1a5/Asct2 transport serine and glutamine [31, 32]. The concentration of serine in human cerebrospinal fluid is higher during infancy than during adult age [33]. The clinical phenotypes of serine-deficiency syndromes include neurological dysfunctions [34]. Glutamine supplementation after birth increases white matter, hippocampus, and brain stem volumes in very preterm children [35]. Moreover, serum amino acid concentrations are higher in neonatal mice than in adult mice [36]. Therefore, the induction of amino acid transporters at the BBB is possible to supply substrate amino acids to maintain brain development during the neonatal period. Nevertheless, future studies to clarify the molecular function and transport direction of the induced amino acid transporters at the neonatal BBB are needed. The expression of Slc1a2/Eaat2 and Slc1a3/Eaat1 were significantly lower in the nBC fraction than in the aBC fraction. This reduction is likely to be due to the lower enrichment of astrocytes in the nBC fraction than in the aBC fraction, as discussed for Gfap in the previous paragraph.

The present network analysis revealed that the expression of proteins relating to the extracellular matrix, including integrin, collagen, and laminin, were altered in the neonatal brain capillaries compared to those in the adult capillaries. Previous transcriptomic and proteomic studies have analyzed isolated brain capillaries at postnatal days 5 and 10 and in adult mice and extracted the extracellular matrix and cell adhesion by pathway analysis of differentially expressed genes and proteins [14]. A previous proteomic study identified 899 proteins at three ages and showed the expression of three collagens and four laminins, which were also detected in the present study. Among these seven proteins, the expression of six proteins (Col4a1, Col6a1, Lama5, Lama2, Lamab2, and Lamac1) between P5 and adults in the previous study was regulated similarly to that between P7 and adults in the present study. The expression of Col1a2 between P10 and adult stages in the previous study was similar to that observed in the present study. Protein expression data for integrins were not provided in the previous study. These results indicated that the expression changes of collagens and laminin were reproduced in the two proteomic studies, and the present study provided further proteome information. The lower expression of laminin and integrin suggested the immatureness of the extracellular matrix of brain microvasculature. The changes in expression in collagen were type-dependent (Fig. 5C). In the kidney, the developmental switching of subtypes is observed in collagen type IV [37]. The results of the present study propose the possibility of the developmental switching of collagen types during the postnatal period.

## Conclusions

We successfully prepared brain capillary fractions from the single frozen brain of a neonatal mouse at postnatal day 7. The developed method can analyze the function and expression of individual neonatal brain capillaries and contribute to the reduced use of animals in experiments. The brain capillaries were enriched in the fraction, which is sufficient for proteome analysis. The protein expression changes between neonatal and adult brain capillaries were comparable to those in previous studies. Furthermore, proteomic analysis revealed that amino acid transporters, including Slc38a5/Snat5 and Slc1a5/Asct2, were upregulated in neonatal brain capillaries. Therefore, the present isolation method will promote the study on the function and expression of neonatal BBB.

## Supplementary Information

**Additional file 1: Table S1.** Proteomic data of the neonatal brain capillaryfractions and whole neonatal brain lysates. **Table S2.** Proteomic data of brain capillary fractions and whole brain lysates from neonatal and adult mice. **Table S3.** Proteomic data of brain capillary-enriched proteins in brain capillary fractions and whole brain lysates of neonatal and adult mice. **Table S4.** Enriched pathways involving 60 proteins constituting the largest network group in Figure 4A.

**Additional file 2: Figure S1.** Distribution of protein intensities and coefficient of varianceof the proteome data in the present study. **Figure S2.** Principal component analysisof the proteome data in the present study. **Figure S3.** Enlarged network map of the 191 differentially expressed brain capillary-enriched proteins shown in Fig. 5A. **Figure S4.** Network map of the 60 proteins constituting the largest network group. **Figure S5.** Comparison of fold changes in protein expression between the neonatal capillary brainand adult brain capillaryfractions between the present and previous studies.

## Data Availability

Raw data files for the proteomic analysis have been deposited in jPOST (http://jpostdb.org; jPOST ID: JPST001978/PXD039296 and JPST002139/PXD041770).

## Abbreviations

aBC: Adult brain capillary
BBB: Blood-brain barrier
BCECs: Brain capillary endothelial cells
BC: Brain capillary
nBC: Neonatal brain capillary
BCA: Bicinchoninic acid
PVDF: Polyvinylidene fluoride
HRP: Horseradish peroxidase
SDS: Sodium dodecyl sulfate
%CV: Percentage of the coefficient of variance

## References

[1] Abbott NJ, Ronnback L, Hansson E (2006). Astrocyte-endothelial interactions at the blood-brain barrier. Nat Rev Neurosci.

[2] Keaney J, Campbell M (2015). The dynamic blood-brain barrier. FEBS J.

[3] Ohtsuki S, Terasaki T (2007). Contribution of carrier-mediated transport systems to the blood-brain barrier as a supporting and protecting interface for the brain; importance for CNS drug discovery and development. Pharm Res.

[4] Pardridge WM (2012). Drug transport across the blood-brain barrier. J Cereb Blood Flow Metab.

[5] Morimoto K, Nagami T, Matsumoto N, Wada S, Kano T, Kakinuma C, Ogihara T (2012). Developmental changes of brain distribution and localization of oseltamivir and its active metabolite Ro 64–0802 in rats. J Toxicol Sci.

[6] Verscheijden LFM, van Hattem AC, Pertijs J, de Jongh CA, Verdijk RM, Smeets B, Koenderink JB, Russel FGM, de Wildt SN (2020). Developmental patterns in human blood-brain barrier and blood-cerebrospinal fluid barrier ABC drug transporter expression. Histochem Cell Biol.

[7] Ohtsuki S, Kikkawa T, Hori S, Terasaki T (2006). Modulation and compensation of the mRNA expression of energy related transporters in the brain of glucose transporter 1-deficient mice. Biol Pharm Bull.

[8] Omori K, Tachikawa M, Hirose S, Taii A, Akanuma SI, Hosoya KI, Terasaki T (2020). Developmental changes in transporter and receptor protein expression levels at the rat blood-brain barrier based on quantitative targeted absolute proteomics. Drug Metab Pharmacokinet.

[9] Cremer JE, Cunningham VJ, Pardridge WM, Braun LD, Oldendorf WH (1979). Kinetics of blood-brain barrier transport of pyruvate, lactate and glucose in suckling, weanling and adult rats. J Neurochem.

[10] Garcia FJ, Sun N, Lee H, Godlewski B, Mathys H, Galani K, Zhou B, Jiang X, Ng AP, Mantero J, Tsai LH, Bennett DA, Sahin M, Kellis M, Heiman M (2022). Single-cell dissection of the human brain vasculature. Nature.

[11] Vanlandewijck M, He L, Mae MA, Andrae J, Ando K, Del Gaudio F, Nahar K, Lebouvier T, Lavina B, Gouveia L, Sun Y, Raschperger E, Rasanen M, Zarb Y, Mochizuki N, Keller A, Lendahl U, Betsholtz C (2018). A molecular atlas of cell types and zonation in the brain vasculature. Nature.

[12] Laporte MH, Chatellard C, Vauchez V, Hemming FJ, Deloulme JC, Vossier F, Blot B, Fraboulet S, Sadoul R (2017). Alix is required during development for normal growth of the mouse brain. Sci Rep.

[13] Lin Y, Wang X, Rose KP, Dai M, Han J, Xin M, Pan D (2020). miR-143 regulates lysosomal enzyme transport across the blood-brain barrier and transforms CNS treatment for mucopolysaccharidosis type I. Mol Ther.

[14] Porte B, Hardouin J, Zerdoumi Y, Derambure C, Hauchecorne M, Dupre N, Obry A, Lequerre T, Bekri S, Gonzalez B, Flaman JM, Marret S, Cosette P, Leroux P (2017). Major remodeling of brain microvessels during neonatal period in the mouse: A proteomic and transcriptomic study. J Cereb Blood Flow Metab.

[15] Ogata S, Ito S, Masuda T, Ohtsuki S (2021). Efficient isolation of brain capillary from a single frozen mouse brain for protein expression analysis. J Cereb Blood Flow Metab.

[16] Nagano H, Ito S, Masuda T, Ohtsuki S (2022). Effect of insulin receptor-knockdown on the expression levels of blood-brain barrier functional proteins in human brain microvascular endothelial cells. Pharm Res.

[17] Masuda T, Tomita M, Ishihama Y (2008). Phase transfer surfactant-aided trypsin digestion for membrane proteome analysis. J Proteome Res.

[18] Mori A, Masuda T, Ito S, Ohtsuki S (2022). Human hepatic transporter signature peptides for quantitative targeted absolute proteomics: selection, digestion efficiency, and peptide stability. Pharm Res.

[19] Demichev V, Messner CB, Vernardis SI, Lilley KS, Ralser M (2020). DIA-NN: neural networks and interference correction enable deep proteome coverage in high throughput. Nat Methods.

[20] Cox J, Hein MY, Luber CA, Paron I, Nagaraj N, Mann M (2014). Accurate proteome-wide label-free quantification by delayed normalization and maximal peptide ratio extraction, termed MaxLFQ. Mol Cell Proteomics.

[21] Szklarczyk D, Gable AL, Nastou KC, Lyon D, Kirsch R, Pyysalo S, Doncheva NT, Legeay M, Fang T, Bork P, Jensen LJ, von Mering C (2021). The STRING database in 2021: customizable protein-protein networks, and functional characterization of user-uploaded gene/measurement sets. Nucleic Acids Res.

[22] Macdonald JA, Murugesan N, Pachter JS (2010). Endothelial cell heterogeneity of blood-brain barrier gene expression along the cerebral microvasculature. J Neurosci Res.

[23] Chasseigneaux S, Moraca Y, Cochois-Guegan V, Boulay AC, Gilbert A, Le Crom S, Blugeon C, Firmo C, Cisternino S, Laplanche JL, Curis E, Decleves X, Saubamea B (2018). Isolation and differential transcriptome of vascular smooth muscle cells and mid-capillary pericytes from the rat brain. Sci Rep.

[24] Bushong EA, Martone ME, Ellisman MH (2004). Maturation of astrocyte morphology and the establishment of astrocyte domains during postnatal hippocampal development. Int J Dev Neurosci.

[25] Sundaram SM, Safina D, Ehrkamp A, Faissner A, Heumann R, Dietzel ID (2019). Differential expression patterns of sodium potassium ATPase alpha and beta subunit isoforms in mouse brain during postnatal development. Neurochem Int.

[26] Tachikawa M, Hirose S, Akanuma SI, Matsuyama R, Hosoya KI (2018). Developmental changes of l-arginine transport at the blood-brain barrier in rats. Microvasc Res.

[27] Sakai K, Shimizu H, Koike T, Furuya S, Watanabe M (2003). Neutral amino acid transporter ASCT1 is preferentially expressed in L-Ser-synthetic/storing glial cells in the mouse brain with transient expression in developing capillaries. J Neurosci.

[28] Zhang Y, Sloan SA, Clarke LE, Caneda C, Plaza CA, Blumenthal PD, Vogel H, Steinberg GK, Edwards MS, Li G, Duncan JA, Cheshier SH, Shuer LM, Chang EF, Grant GA, Gephart MG, Barres BA (2016). Purification and characterization of progenitor and mature human astrocytes reveals transcriptional and functional differences with mouse. Neuron.

[29] Zhang Y, Chen K, Sloan SA, Bennett ML, Scholze AR, O'Keeffe S, Phatnani HP, Guarnieri P, Caneda C, Ruderisch N, Deng S, Liddelow SA, Zhang C, Daneman R, Maniatis T, Barres BA, Wu JQ (2014). An RNA-sequencing transcriptome and splicing database of glia, neurons, and vascular cells of the cerebral cortex. J Neurosci.

[30] Hosoya K, Sugawara M, Asaba H, Terasaki T (1999). Blood-brain barrier produces significant efflux of L-aspartic acid but not D-aspartic acid: in vivo evidence using the brain efflux index method. J Neurochem.

[31] Utsunomiya-Tate N, Endou H, Kanai Y (1996). Cloning and functional characterization of a system ASC-like Na+-dependent neutral amino acid transporter. J Biol Chem.

[32] Nakanishi T, Sugawara M, Huang W, Martindale RG, Leibach FH, Ganapathy ME, Prasad PD, Ganapathy V (2001). Structure, function, and tissue expression pattern of human SN2, a subtype of the amino acid transport system N. Biochem Biophys Res Commun.

[33] Fuchs SA, Dorland L, de Sain-van der Velden MG, Hendriks M, Klomp LW, Berger R, de Koning TJ. D-serine in the developing human central nervous system. Ann Neurol. 2006;60(4):476–80. 10.1002/ana.20977 17068790

[34] El-Hattab AW (2016). Serine biosynthesis and transport defects. Mol Genet Metab.

[35] de Kieviet JF, Oosterlaan J, Vermeulen RJ, Pouwels PJ, Lafeber HN, van Elburg RM (2012). Effects of glutamine on brain development in very preterm children at school age. Pediatrics.

[36] Helman A, Cangelosi AL, Davis JC, Pham Q, Rothman A, Faust AL, Straubhaar JR, Sabatini DM, Melton DA. A nutrient-sensing transition at birth triggers glucose-responsive insulin secretion. Cell Metab. 2020;31(5):1004–16 e5. 10.1016/j.cmet.2020.04.004 PMC7480404 32375022

[37] Miner JH, Sanes JR (1994). Collagen IV alpha 3, alpha 4, and alpha 5 chains in rodent basal laminae: sequence, distribution, association with laminins, and developmental switches. J Cell Biol.

