# Supplementary material for: Development of a method for isolating brain capillaries from a single neonatal mouse brain and comparison of proteomic profiles between neonatal and adult brain capillaries
Source: Fluids Barriers CNS. 2023 Jun 23;20:50. doi: 10.1186/s12987-023-00449-w (PMC10290343; doi:10.1186/s12987-023-00449-w)
Supplement: Supplementary file 2 — Additional file 2: Figure S1. Distribution of protein intensities and coefficient of varianceof the proteome data in the present study. Figure S2. Principal component analysisof the proteome data in the present study. Figure S3. Enlarged network map of the 191 differentially expressed brain capillary-enriched proteins shown in Fig. 5A. Figure S4. Network map of the 60 proteins constituting the largest network group. Figure S5. Comparison of fold changes in protein expression between the neonatal capillary brainand adult brain capillaryfractions between the present and previous studies. [file 12987_2023_449_MOESM2_ESM.docx]

**Fluids and Barriers of the CNS**

**Additional Information**

**Development of an isolation method for brain capillaries from a single neonatal mouse brain**

Yudai Hamada^1^, Seiryo Ogata^2^, Takeshi Masuda^1,3^, Shingo Ito^1,3^, Sumio Ohtsuki^1,3^

^1^Department of Pharmaceutical Microbiology, School of Pharmacy, Kumamoto University, 5-1 Oe-honmachi, Chuo-ku, Kumamoto 862-0973, Japan

^2^Department of Environmental Medicine and Molecular Toxicology, Tohoku University Graduate School of Medicine, 2-1 Seiryo-machi, Aoba-ku, Sendai 980-8575, Japan.

^3^Department of Pharmaceutical Microbiology, Faculty of Life Sciences, Kumamoto University, 5-1 Oe-honmachi, Chuo-ku, Kumamoto 862-0973, Japan

^*^Corresponding author: Sumio Ohtsuki, Ph.D.

Department of Pharmaceutical Microbiology, Faculty of Life Sciences, Kumamoto University, 5-1 Oe-honmachi, Chuo-ku, Kumamoto 862-0973, Japan

TEL: +81-96-371-4323; FAX: +81-96-371-4329; E-mail: sohtsuki@kumamoto-u.ac.jp

**(Additional_Tables.xlsx)**

**Table S1 Proteomic data of the neonatal brain capillary (nBC) fractions and whole neonatal brain lysates**

**Table S2 Proteomic data of brain capillary fractions and whole brain lysates from neonatal and adult mice**

**Table S3 Proteomic data of brain capillary-enriched proteins in brain capillary fractions and whole brain lysates of neonatal and adult mice**

**Table S4 Enriched pathways involving 60 proteins constituting the largest network group in Figure 4A**

**
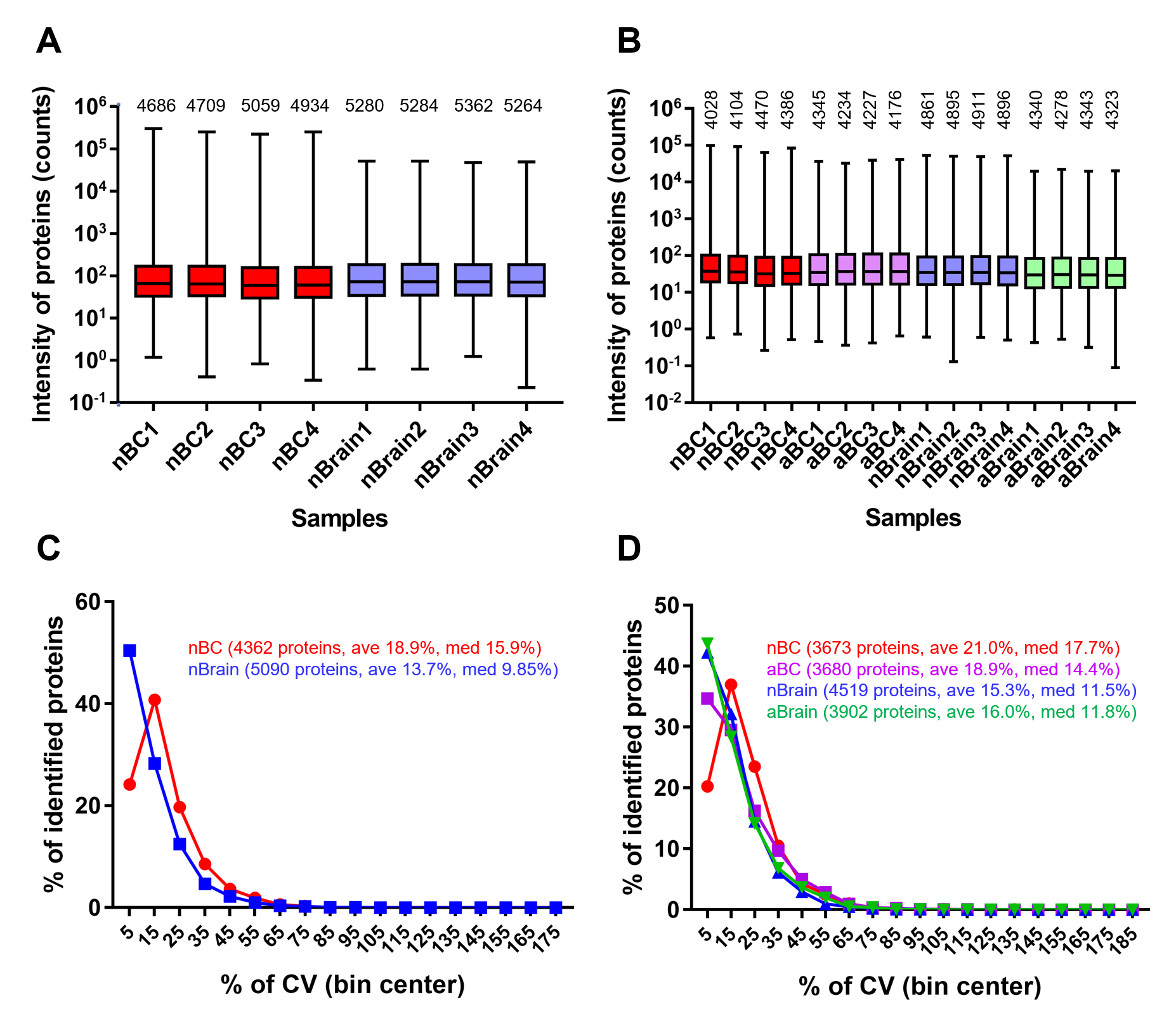
**

**Figure S1 Distribution of protein intensities and coefficient of variance (CV) of the proteome data in the present study**

(A and B) Distribution of intensities of proteins identified in each sample: neonatal brain capillary (nBC) and adult brain capillary (aBC) fractions and lysates of the neonatal brain (nBrain) and adult brain (aBrain). Proteome data of neonates (A) and that of neonates and adults (B). The number following the sample name represents the number of replicates. The numbers at the top of each plot indicate the number of proteins identified. The centerline indicates the median value, and the box contains the 25th and 75th percentiles. Whiskers indicate the largest and smallest values. (C and D) Distribution of the %CV in each sample group. The %CV was calculated for proteins identified in all four replicates. Proteome data of neonates (C), neonates and adults (D). The numbers in parentheses represent the number of identified proteins, the average, and the median.

**
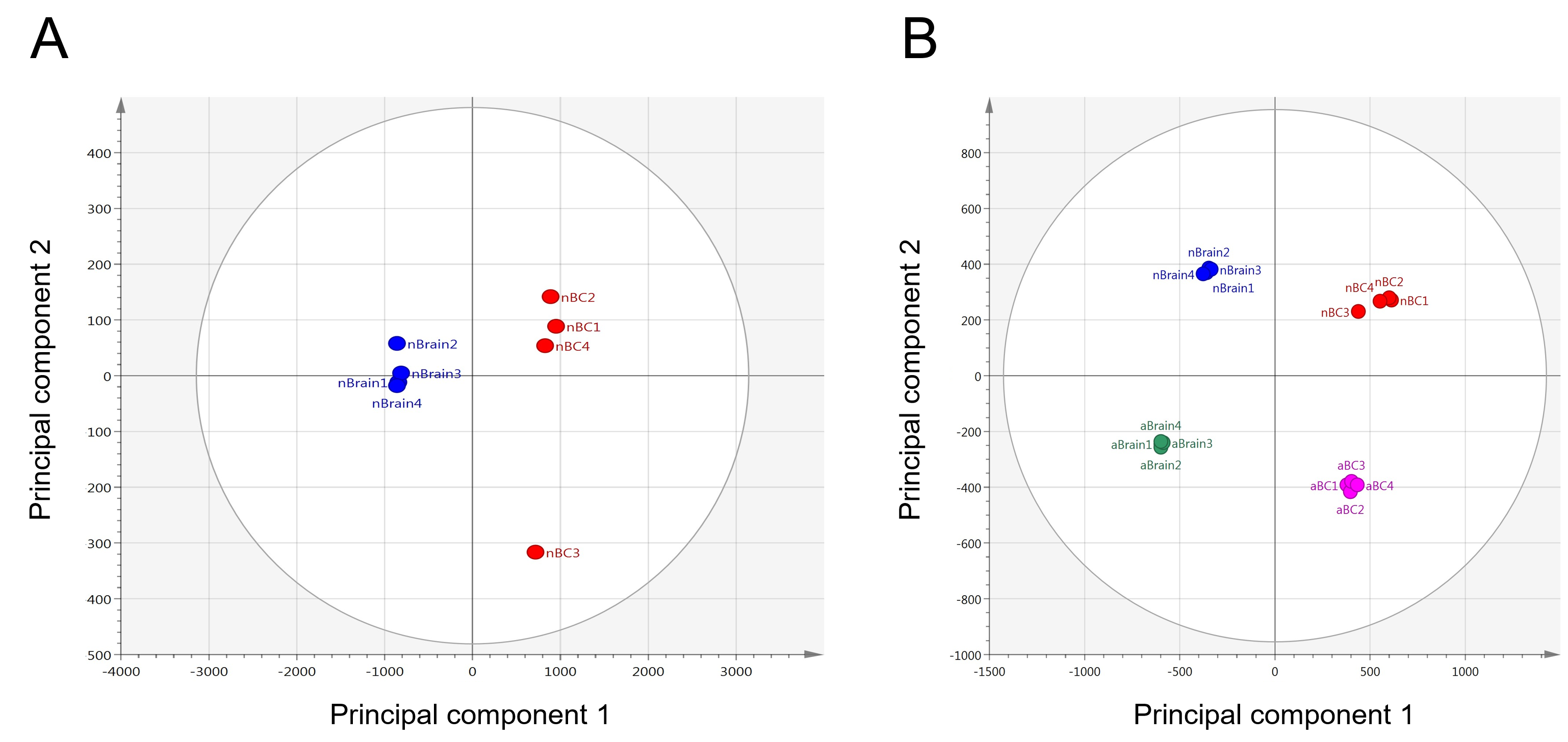
**

**Figure S2 Principal component analysis (PCA) of the proteome data in the present study.**

Score plot of proteome data of neonates (A) and neonates and adults (B) containing data from neonatal brain capillary (nBC) and adult brain capillary (aBC) fractions and lysates of neonatal brain (nBrain) and adult brain (aBrain). The number following the sample name represents the number of replicates. PCA was performed using SIMCA14 (Sartorius, Gottingen, Germany). The variables were centered and scaled to Pareto Variance.

**
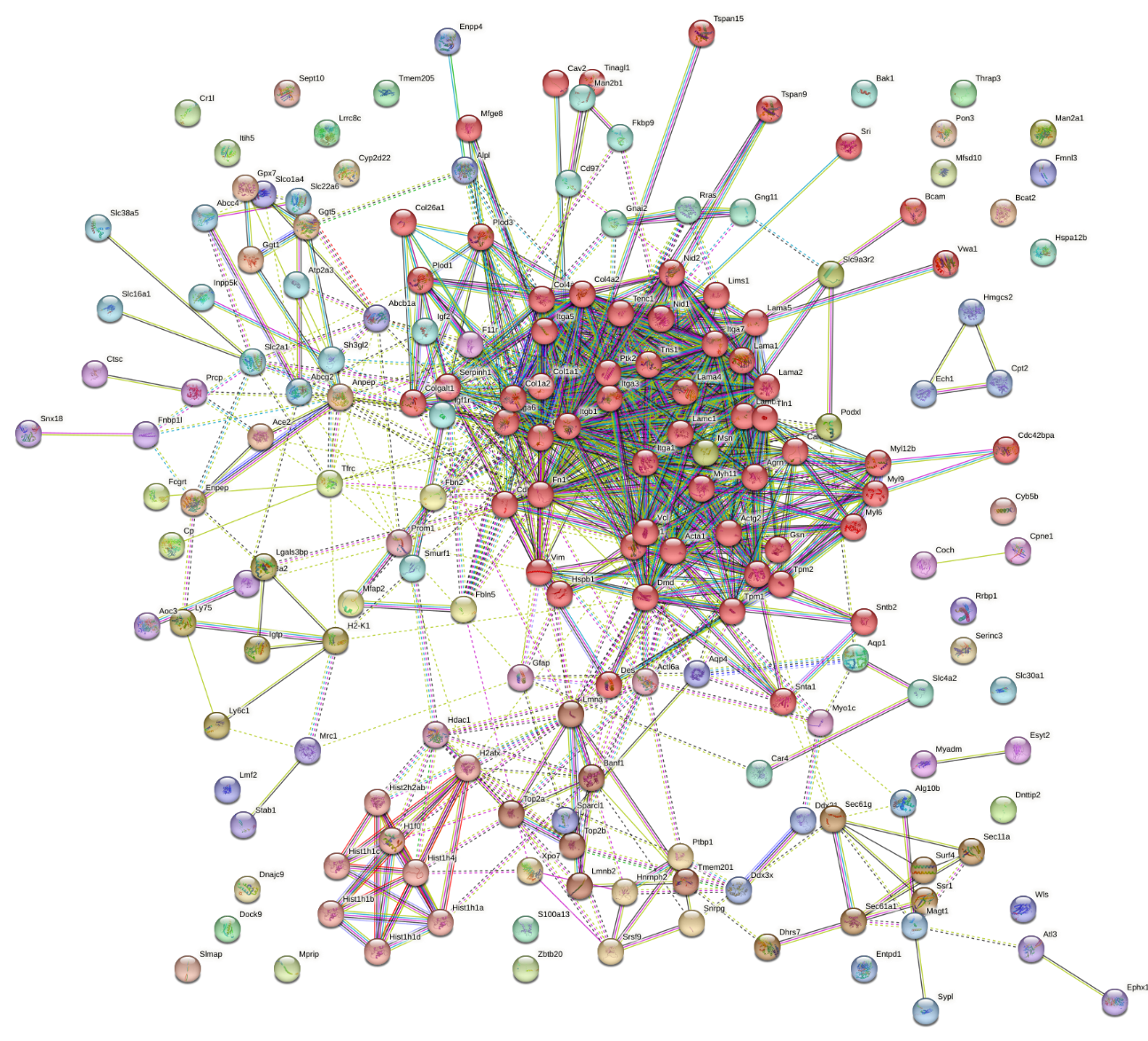
**

**Figure S3 Enlarged network map of the 191 differentially expressed brain capillary (BC)-enriched proteins shown in Fig. 5A.**

**
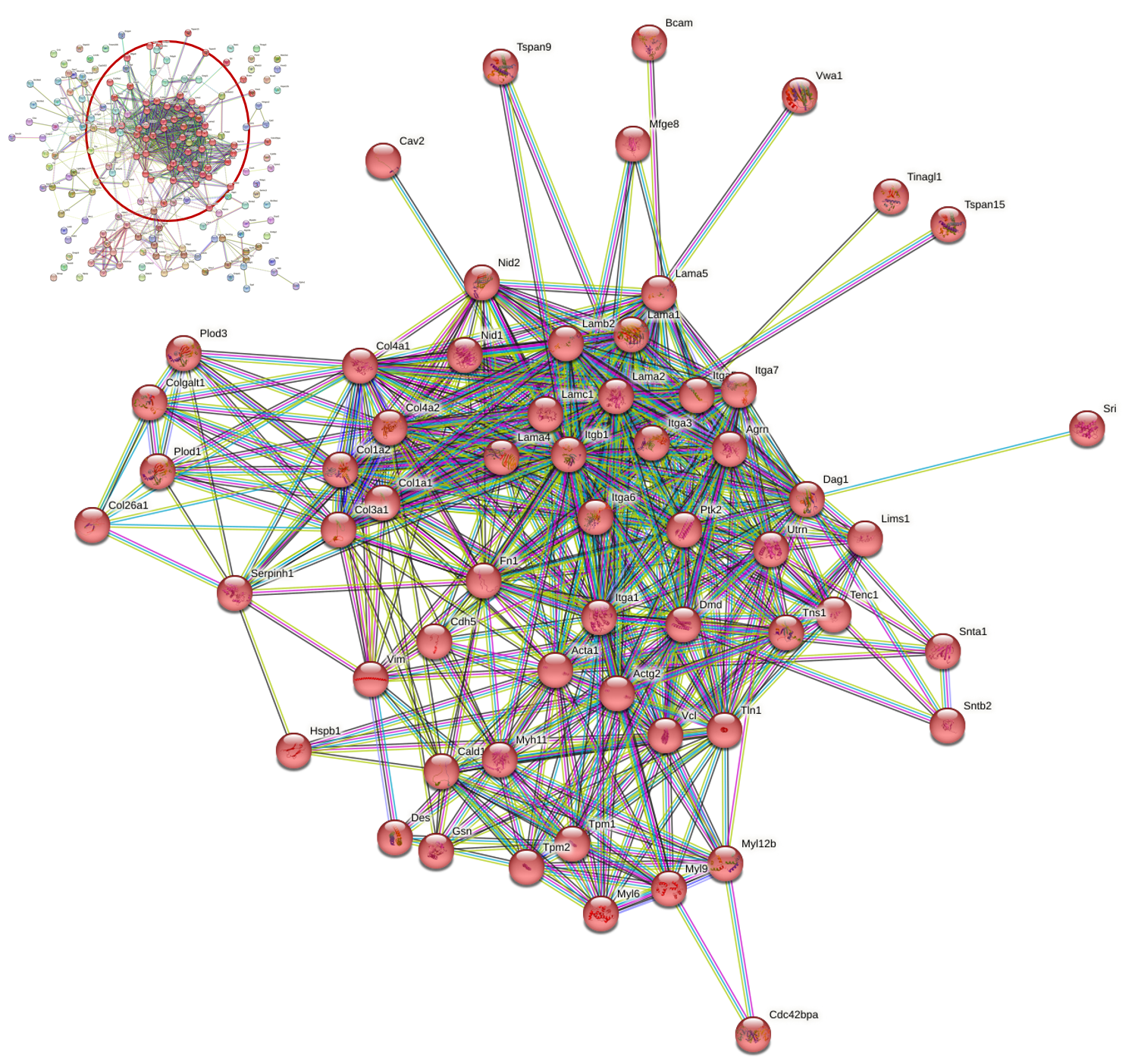
**

**Figure S4 Network map of the 60 proteins constituting the largest network group.**

**
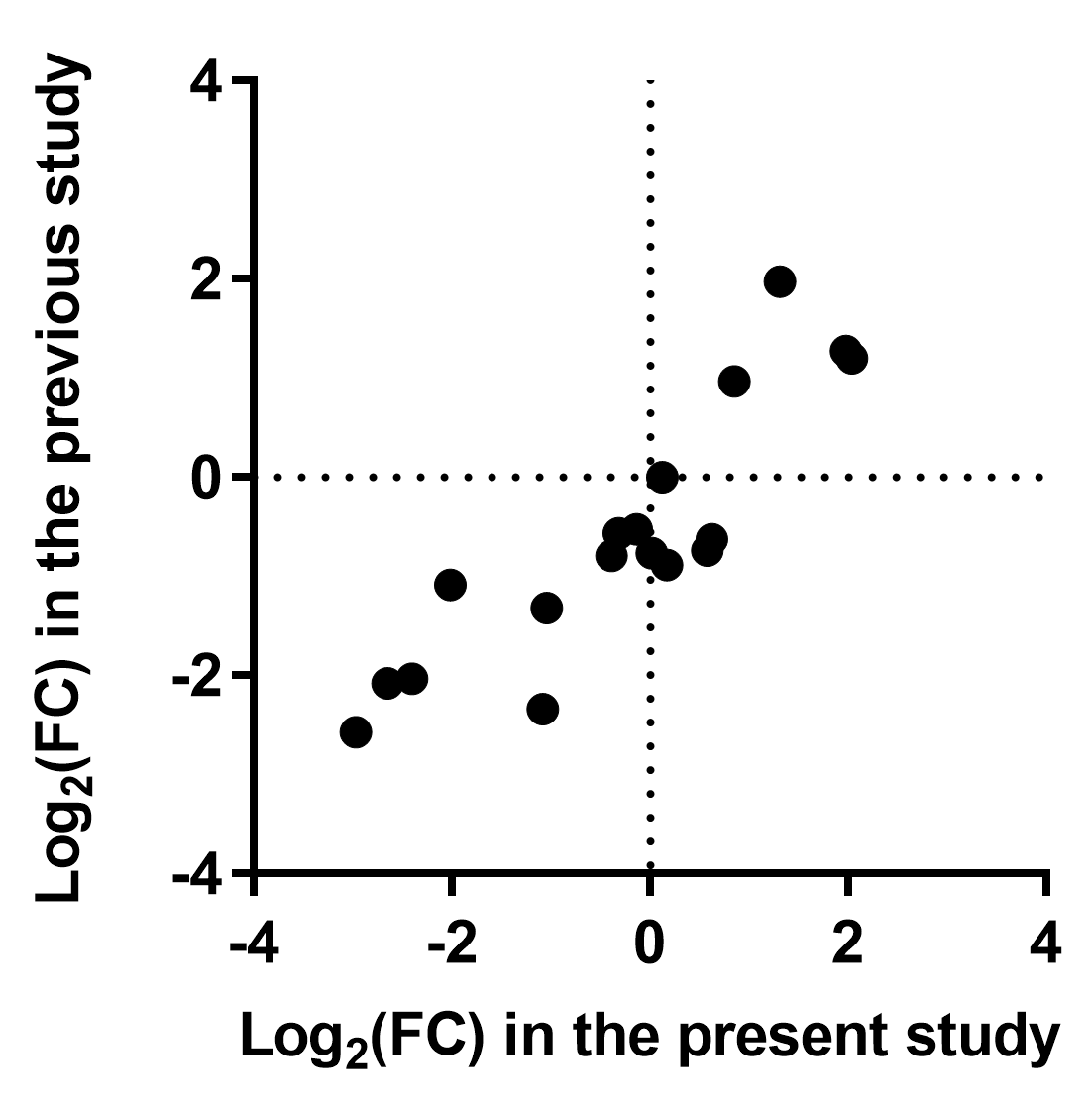
**

**Figure S5 Comparison of fold changes in protein expression between the neonatal brain capillary (nBC) and adult brain capillary (aBC) fractions between the present and previous studies.**

Fold changes (FC) were calculated from the data in our present mouse study and the previous rat study reported by Omori *et al* (8).
